# Small molecules of herbal origin for osteoarthritis treatment: in vitro and in vivo evidence

**DOI:** 10.1186/s13075-022-02785-y

**Published:** 2022-05-11

**Authors:** Penghui Zhang, Kaihu Li, Amir Kamali, Reihane Ziadlou, Paras Ahmad, Xinluan Wang, R. Geoff Richards, Mauro Alini, Valentina Basoli, Zhen Li, Sibylle Grad

**Affiliations:** 1 AO Research Institute Davos, Davos, Switzerland; 2 Department of Orthopaedic Surgery, The Seventh Affiliated Hospital, Sun Yat-sen University, Shenzhen, China; 3 Department of Orthopaedics, Xiangya Hospital of Central South University, Changsha, China; 4 Translational Medicine R&D Center, Shenzhen Institute of Advanced Technology, Chinese Academy of Sciences, Shenzhen, China; 5 Department of Health Sciences and Technology, ETH Zurich, Zürich, Switzerland

**Keywords:** Traditional Chinese medicine, Herbal medicine, Herbal extraction, Therapeutic target, Compound delivery, Inflammation, Anti-inflammatory, Anabolic, Anti-catabolic

## Abstract

Osteoarthritis (OA) is one of the most common musculoskeletal degenerative diseases and contributes to heavy socioeconomic burden. Current pharmacological and conventional non-pharmacological therapies aim at relieving the symptoms like pain and disability rather than modifying the underlying disease. Surgical treatment and ultimately joint replacement arthroplasty are indicated in advanced stages of OA. Since the underlying mechanisms of OA onset and progression have not been fully elucidated yet, the development of novel therapeutics to prevent, halt, or reverse the disease is laborious. Recently, small molecules of herbal origin have been reported to show potent anti-inflammatory, anti-catabolic, and anabolic effects, implying their potential for treatment of OA. Herein, the molecular mechanisms of these small molecules, their effect on physiological or pathological signaling pathways, the advancement of the extraction methods, and their potential clinical translation based on in vitro and in vivo evidence are comprehensively reviewed.

## Background

Globally, osteoarthritis (OA) is one of the most commonly occurring disorders of articular joints [1–3], which typically affects the knees, spine, hands, hips, and feet [4]. The incidence and prevalence reported in epidemiological studies fluctuate widely, as the data may be based on different diagnostic criteria (clinical, radiographic, or pathological OA), various joint locations, and diverse patient populations [5]. The reported prevalence of OA thus ranges from 12.3 to 21.6% [6, 7]. Women are at a higher risk for developing OA as compared to men [2, 7]. The risk further escalates with age, with OA usually occurring in the fourth or fifth decade of life [8, 9]. Furthermore, OA prevalence varies widely in different regions (e.g., developing or developed countries, rural or urban areas) [10–12].

Joint pain, joint swelling, locomotion restriction, and joint stiffness are the principal symptoms of OA, while other symptoms like crepitus and joint deformation are also encountered. Recurrent and progressive joint pain, being relieved by rest and worsened with joint exercise, is the most problematic symptom. Although the origin of pain in OA is not fully understood, it may arise from mechanoreceptors and nociceptive fibers in the synovium, capsule, subchondral bone, tendons, periosteum, or ligaments [13].

Recent studies report that the disease burden of OA is comparable to that of rheumatoid arthritis [14, 15]. Globally, a 75% rise was observed in OA-related years lived with disabilities (YLDs) from 1990 to 2013, with OA accounting for 2.4% of all YLDs [16]. Hence, OA represents the third fastest increasing condition related to disability, following dementia and diabetes [16, 17]. In the USA (2010), approximately 10% of all ambulatory care visits were to diagnose arthritis and other rheumatic diseases, 58% of which were estimated to be related to symptomatic OA [18]. The economic burden due to OA comprises three parts: indirect costs such as loss of productivity and disability payments; direct costs like hospital resources, conservative treatment, and research; and intangible costs caused by mental illness and reduced life quality.

## Pathophysiology and treatment of osteoarthritis

Multiple pathological processes jointly contribute to the development of OA, and various phenotypes are thus formed [19]. The risk factors for OA development include age, trauma, obesity, innate immunity, metabolic diseases, systemic inflammatory conditions, and genetic predisposition [12, 20]. Currently, low-grade inflammation is the primary focus of OA pathophysiology because the reported risk factors may lead to chronic inflammatory conditions [21]. Another pathological process that might lead to OA is the metabolically triggered inflammation [22]. Metabolic disturbance of nutrients and metabolites leads to oxidative stress and chronic inflammation by inducing adipocytes to release adipokines, C-reactive protein (CRP), complement components, cytokines, and other pro-inflammatory mediators, which may result in cartilage and surrounding tissue destruction. Pro-catabolic and pro-inflammatory mediators, along with mechanical and oxidative stressors, deteriorate the performance and life cycle of chondrocytes, induce hypertrophy, and ultimately make them prone to degenerative responses [19].

**Fig. 1:**
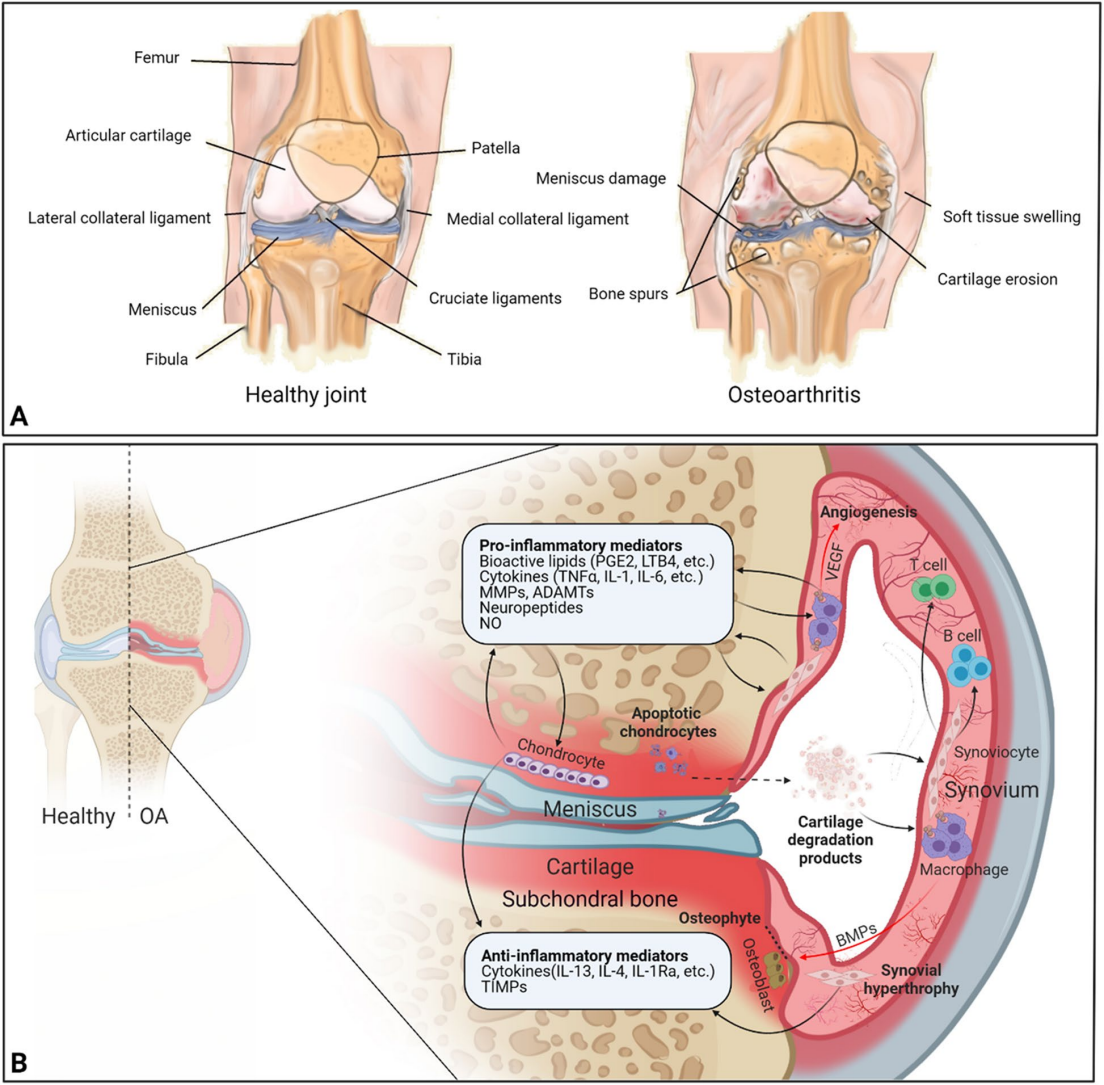
Overview of the healthy and osteoarthritic joint along with the pathophysiology of OA. **A** Healthy joint is depicted: intact cartilage with no fissures and synovial inflammation signs. Osteoarthritis is characterized by soft tissue swelling, osteophyte formation, meniscus deterioration, and degeneration of cartilage. **B** Cartilage breakdown products are released from the damaged cartilage tissue into the joint space, which are phagocytosed by the synovial cells and infiltrated macrophages, intensifying synovial inflammation. In the inflamed synovium, pro-inflammatory and catabolic mediators are produced by the activated synovial cells that cause overproduction of the proteolytic enzymes, establishing a positive feedback loop. The activated synovial B cells, T cells, and infiltrating macrophages amplify the inflammatory response. To neutralize this inflammatory response, anti-inflammatory cytokines are produced by the synoviocytes and chondrocytes. Furthermore, the inflamed macrophages contribute to the synovial angiogenesis and osteophyte formation via VEGF and BMPs release, respectively. Panel **B** is adapted from Sellam et al. [27] with permission, copyright 2010, Springer Nature. (The figure was prepared with Biorender). ADAMTS, a disintegrin and metalloproteinase with thrombospondin motifs; BMP, bone morphogenetic protein; IL, interleukin; IL-1Ra, IL-1 receptor antagonist; LTB4, leukotriene B4; MMP, matrix metalloproteinase; NO, nitric oxide; OA, osteoarthritis; PGE2, prostaglandin E2; TIMP, tissue inhibitor of metalloproteinase; TNF, tumor necrosis factor; VEGF, vascular endothelial growth factor

While autophagy is reported to preserve chondrocytes, and its loss may contribute to OA development [23], apoptosis also takes place amidst OA development and endochondral ossification [24]. Cartilage degradation products, mainly cartilage wear particles [25], may lead to hyperplasia and hypertrophy of synovial fibroblasts/fibrocytes, which can induce the activation of B and T cells to amplify the inflammation [26, 27]. The inflammatory responses also lead to the activation of macrophages, which release several pro-inflammatory mediators, matrix metalloproteinases, bioactive lipids, neuropeptides, and growth factors. A feedback of cartilage breakdown and synovial inflammation is subsequently induced. However, chondrocytes and synovial tissues also release anti-inflammatory mediators, such as IL-4, IL-10, and IL-13 to modulate the inflammatory responses. Besides, the inflamed synovium can induce angiogenesis and osteophyte formation (Fig. 1).

Currently, there is no licensed drug with valid disease-modifying activity against OA. Hence, the management of OA is targeted at improving disability, pain, and life quality with pharmacologic and non-pharmacologic therapies. The most effective and safest treatment for knee/hip OA is physical exercise/activity coupled with self-management approaches [18, 28]. All OA management guidelines [29–31] advocate the promotion of healthy weight and self-management, along with a combination of aerobic and strengthening exercises [32] as core management approaches. Furthermore, systemic or local non-steroidal anti-inflammatory drugs (NSAIDs), intra-articular hyaluronan injection, or intra-articular corticosteroids are frequently administered [28], though with poor long-term outcomes.

Joint replacement is recommended when non-surgical methods cannot control OA symptoms. Nonetheless, joint replacement is not a cure for OA; around 20~30% of knee and hip replacement patients report no or little improvement and are not satisfied with the results of their surgery 1 year post-joint replacement [33, 34]. Worldwide, while scientists continue to work towards a better understanding of the predisposing factors for poor results following knee/hip joint replacement, there remains an urgent need to recognize effective and safe non-surgical modalities to prevent and treat OA.

The use of herbal medicine for analgesic and anti-inflammatory therapy of joint diseases has been practiced for a long time in the form of phytotherapeutic drugs in both the eastern and western worlds. Herbal compounds extracted from traditional Chinese medicine (TCM) plants have been applied for the treatment of multiple diseases, with artemisinin used for the treatment of malaria as the most well-known example (2015 Nobel prize by Youyou Tu). More recently, the chondroprotective and inflammation modulatory effects of herbal extracts or their individual small molecule compounds have been evaluated in preclinical in vitro and in vivo studies [35]. Several herbal compounds with potential disease-modifying activities have been identified. Nevertheless, only few clinical studies have been documented so far. The aim of this literature review is to provide an overview of promising small-molecule compounds with herbal origin and their mechanism of action, as described in preclinical investigations for the prevention or treatment of OA and related pathologies.

## Extraction of small molecules from medicinal herbs

The discovery of new natural compounds is of great significance for drug exploitation and development in the treatment of OA. The formulations used in TCM are extremely complex and contain a variety of effective ingredients. To improve drug efficacy, reduce side effects, and explore the pharmacological mechanism, it is important to extract the components and purify them to obtain effective single compounds. In recent years, some new technologies and methods have been developed to extract and isolate the active ingredients of TCM herbs. The application of these technologies not only helps to improve the extraction rate and the purity of active ingredients, but also facilitates the study of structure, pharmacology, and efficacy.

Supercritical fluid extraction is a technology that uses the supercritical fluid as an extractant to isolate and separate the medicinal components in TCM. This method has the advantages of high extraction efficiency, high purification, easy operation, and absence of residual solvent contamination [36, 37]. Supercritical fluid extraction has widely been used in a variety of TCM herbs. Using this method, Huang et al. extracted and separated psoralen and isopsoralen from *Psoralea corylifolia* [38]. Furthermore, ginkgolic acids from the epicarp of *Ginkgo biloba*, tautomeric 7-epimeric spiro oxindole alkaloids from *Uncaria macrophylla*, coumarin from radix *Angelicae dahuricae*, and chrysophanol from *Rheum palmatum* were extracted by supercritical fluid extraction [36, 37, 39, 40].

Ultrasound-assisted extraction is an extraction method with the assistance of ultrasonic energy. It mainly uses the cavitation of ultrasonic waves to accelerate the extraction of active ingredients from plants. In addition, secondary effects of ultrasonic waves, including mechanical vibration, emulsification, diffusion, crushing, and chemical effects, can also accelerate the diffusion-based release of the ingredients to be fully mixed with the solvent, which helps to improve the yield of the effective ingredients. Moreover, the ultrasonic crushing process is a physical process without chemical reaction during the extraction, maintaining the bioactivity of components. Ultrasound-assisted extraction is mainly used for the isolation of plant alkaloids, glycosides, and phenolic compounds [41–51].

The high-speed countercurrent chromatography (HSCCC) technique was developed from countercurrent chromatography and is characterized by high purity, good reproducibility, high separation efficiency, and low solvent usage in the extraction of medicinal herbs [52, 53]. It is particularly suitable for the separation of polar compounds. Fang et al. isolated and purified three ecdysteroids with anti-inflammatory effects from the stems of *Diploclisia glaucescens* by the HSCCC technique [54]. Nine compounds with over 95% purity were obtained by the HSCCC technique from the root of *Adenophora tetraphlla* [53]. HSCCC was reported as a powerful tool to separate the main compounds from the rhizome of *Smilax glabra* and was valuable for the preparative separation of compounds with broad *K*-values and similar structures [55].

Many other extraction methods were also used to extract the small molecules from medicinal herbs due to the extreme complexity of their chemical compositions. As a conventional technique, solvent extraction faces some limitations such as using too much organic solvents, losing some volatile compounds, the possibility of leaving toxic solvent residues in the extract, and low extraction efficiency [56]. Microwave-assisted extraction which heats the solvent in contact with the sample by means of microwave energy is an alternative way of increasing the efficiency of conventional extraction methods to extract target compounds from various raw materials [57]. Ultimately, if the effective constituent in the herbs is identified, successful biochemical synthesis is also a valid option due to its high efficiency.

## Therapeutic effects of small molecules: anti-inflammatory, anti-catabolic, and anabolic activity

Inflammatory responses and cartilage matrix degradation play a critical role in the progression of OA. Thus, many scholars have studied and explored small molecules extracted from TCM for interfering with inflammation and degeneration or have modified their structure to make them more effective against OA (Table 1). Curcumin, a polyphenol separated from thye rhizomes of *Turmeric* and *Curcuma longa*, was shown to reduce inflammation in knee OA in rats through blocking the TLR4/MyD88/NF-κB signaling pathway [60]. Paeonol, one of the active compounds found in *Paeonia lactiflora* pallas, *Cynanchum paniculatum*, and *Paeonia suffruticosa*, had anti-inflammatory efficacy on IL-1β-stimulated human primary chondrocytes by downregulating the expression of IL-6 and tumor necrosis factor (TNF)-α, thereby inhibiting phosphorylation of IκBα and activation of nuclear factor-κB (NF-κB). It also prevented cartilage matrix degradation by reducing the expression of matrix metalloproteinase (MMP)-3 and MMP-13 and preserving the expression of type II collagen [83]. In human osteoarthritic chondrocytes, vanillic acid showed significant anti-inflammatory properties, which were attributed to an inhibition of phosphorylation in the NF-κB signaling pathway [84].

**Table 1:** Effect of small molecules extracted from traditional Chinese medicine on osteoarthritis

Compound (TCM)	Model used	Study design	Delivery and dose	Effects/targets (downregulated↓, upregulated↑)				Pathway/mechanism	Reference
				Anti-inflammation	Anti-catabolism	Anabolism	Others		
Anemonin	h-chondrocytes	Pretreated for 1 h before 10 ng/mL IL-1β treatment for 24 h	10 μM	IL-1β↓, IL-6↓, IL-8↓	MMP-3↓, MMP-13↓	Aggrecan↑	Collagen X↓, p-IKKα/β↓, p-p65↓	NF-κB	Wang et al. [58]
	h-articular cartilage	10 ng/mL IL-1β with/without Anemonin for 4 days	10 μM		MMP-13 positive cells↓, GAG release↓, proteoglycan loss↓	Collagen II↑	Collagen X positive cells↓		
	Mouse	DMM-induced OA for 12 weeks	2 mg/kg (i.a.)		MMP-13↓, ADAMTS-5↓, damage of articular cartilage structure↓	Aggrecan↑, proteoglycan↑	Chondrocyte hypertrophy↓, chondrocyte apoptosis↓; p-p65↓		
Resveratrol	Porcine chondrocytes	Pretreated for 24 h before 100 μg/mL AGEs treatment for 24 h	25, 50, 75, 100 μM	iNOS↓, COX-2↓, NO↓, PGE2↓	MMP-13↓	Collagen II↑	DNA-binding activity of NF-κB↓, IκBα level↑ in a dose-dependent manner; p-IKKα/β, p-ERK↓; NF-κB and AP-1 transcriptional activity↓; JNK activity↓ in a dose-dependent manner	NF-κB and AP-1	Liu et al. [59]
	Porcine cartilage	Pretreated for 24 h before 100 μg/mL AGEs treatment for 72 h	50, 100 μM		Proteoglycan release↓, cleavage products of aggrecan↓				
Curcumin	Rat	MIA-induced OA for 2 weeks	200 mg/kg (i.p.)	Inflammatory cells infiltration↓; IL-6↓, IL-1β↓, and TNF-α↓ in the synovial fluid	Orderly arranged chondrocytes with a smoother articular surface; Mankin score↓		Joint diameter in OA rats↓; paw withdrawal threshold↑; TLR4↓, MyD88↓, p-IκBα↓, NF-κB↓	TLR4/MyD88/NF-κB	Zhang et al. [60]
Amurensin H	Rat chondrocytes	Pretreated for 2 h before 10 ng/mL IL-1β treatment for 24 h	2, 4, 8 μM	NO↓, iNOS↓, PGE2↓, COX-2↓, IL-6↓, IL-17↓, and TNF-α↓ in a dose-dependent manner	MMP-9↓ and MMP-13↓ in a dose-dependent manner	Collagen II↑, GAG↑ in a dose-dependent manner	Mitochondrial ROS↓ in a dose-dependent manner; TLR4↓, p-Syk↓ and TRAF6↓ in a time-dependent manner; p-p65↓ and nuclear translocation↓	TLR4/Syk/NF-κB	Ma et al. [61]
	Mouse	ACLT-induced OA for 2 weeks	10, 20 mg/kg (i.g)		cartilage loss↓, OA course↓				
Paeonol	h-OA chondrocytes	Pretreated for 2 h before 10 ng/mL IL-1β treatment for 24 h	12.5, 25, 50 μM	NO↓, iNOS↓, COX-2↓, and PGE2↓ in a dose-dependent manner	MMP-1↓, MMP-3↓, MMP-13↓ in a dose-dependent manner		p-p65↓, p-IκB↓ and p-PI3K↓, p-AKT↓	PI3K/AKT/NF-κB	Lou et al. [62]
	Mouse	DMM-induced OA for 8 weeks	30 mg/kg (i.p.)		OARSI score↓				
Nobiletin	h-OA chondrocytes	Pretreated for 2 h before 10 ng/mL IL-1β treatment for 1 h	10, 20, 40, 80 μM	iNOS↓, COX-2↓, PGE2↓, NO↓, IL-6↓, and TNF-α↓ in a dose-dependent manner	MMP-13↓ and ADAMTS-5↓ in a dose-dependent manner	Aggrecan↑ and collagen II↑ in a dose-dependent manner	p-p65↓, p-IκB↓, p-PI3K↓, p-AKT↓, and NF-κB promoter luciferase activity↓ in a dose-dependent manner	PI3K/AKT/NF-κB	Xie et al. [63]
	Mouse	DMM-induced OA for 8 weeks	20 mg/kg (i.g)		Protected the structure of articular cartilage and maintained the proteoglycan; OARSI score↓		Subchondral bone thickness↓		
Trans-cinnamaldehyde	SW1353 cell line h-OA chondrocytes	Pretreated for 2 h before 10 ng/mL IL-1β treatment for 6 h	2, 5, 10 μg/mL		MMP-1↓, MMP-3↓, MMP-13↓, ADAMTS-4↓, and ADAMTS-5↓ in a dose-dependent manner		p-IκB𝛼↓, p-p38↓, p-JNK1/2↓ in a dose-dependent manner	NF-κB and p38-JNK	Xia et al. [64]
	Rat	MIA-induced OA for 4 weeks	50 mg/kg (i.p.)		More abundant matrix and smoother superficial zone of the cartilage; OARSI score↓				
Ligustrazine	h-OA chondrocytes	Co-treated with 5 ng/mL IL-1β for 24 h	0.5, 1, 2 μM	IL-1↓, IL-6↓, TNF-α↓	MMP-13↓	Collagen II↑, aggrecan↑, GAG↑	SOD1↑, SOD2↑, SOX9↑, ROS production↓, and MDA↓; nuclear p65↓, p-IκBα↓, and cytoplasmic p65↑; cell apoptosis↓	SOX9/NF-κB	Yu et al. [65]
	Knee OA patients	5 weeks	25, 50, 100 μg/L (po)	IL-1↓, IL-6↓, and TNF-α↓of the joint effusions in a dose-dependent manner			SOD↑ and MDA↓ level of the joint effusions in a dose-dependent manner		
Piperine	h-OA chondrocytes	Pretreated for 2 h before 5 ng/mL IL-1β treatment for 24 h	10, 50, 100 μg/mL	NO↓, PGE2↓, iNOS↓, and COX-2↓ in a dose-dependent manner	MMP-3↓ and MMP-13↓ in a dose-dependent manner		Nuclear p65↓, p-IκBα↓, p-NF-κB↓	NF-κB	Ying et al. [66]
Crocin	Rabbit chondrocytes	Pretreated for 1 h before 5 ng/mL IL-1β treatment for 24 h	5, 25, 50, 100 μM		MMP-1↓, MMP-3↓, MMP-13↓		IκBα↑, NF-κB-dependent transcriptional activity↓	NF-κB	Ding et al. [67]
	Rabbit	ACLT-induced OA	5, 100 μM (i.a)		MMP-1↓, MMP-3↓, MMP-13↓; cartilage degradation↓	Mankin score↓			
Leonurine	Mouse chondrocytes	Pretreated for 2 h before 10 ng/mL IL-1β treatment for 24 h	5, 10, 20 μM	NO↓, PGE2↓, IL-6↓, TNF-α↓, COX-2, and iNOS↓ in a dose-dependent manner	MMP-3↓, MMP-13↓, ADAMTS-5↓	Aggrecan↑, collagen II↑	p-p65↓, p-IκB↓ in a dose-dependent manner	NF-κB	Yin et al. [68]
	Mouse	DMM-induced OA for 6 weeks	30 mg/kg (i.p)		OARSI score↓		Subchondral bone thickness↓, synovitis↓, fibrous cartilage↓		
Isofraxidin	h-OA chondrocytes	Pretreated for 2 h before 10 ng/mL IL-1β treatment for 24 h	1, 10, 50 μM	NO↓, PGE2↓, COX-2, and iNOS↓ in a dose-dependent manner	MMP-1↓, MMP-3↓, MMP-13↓, ADAMTS-4↓, ADAMTS-5↓	Aggrecan↑, collagen II↑	p-p65↓, p-IκB↓	NF-κB	Lin et al. [69]
Juglanin	h-OA chondrocytes	Pretreated for 2 h before 10 ng/mL IL-1β treatment for 24 h	10, 20, 40 μM	NO↓, PGE2↓, COX-2, iNOS↓, TNF-α↓, and IL-6↓ in a dose-dependent manner	MMP-1↓, MMP-3↓, MMP-13↓, ADAMTS-4↓, ADAMTS-5↓		p-p65↓, p-IκB↓	NF-κB	Chen et al. [70]
Scutellarin	Mouse chondrocytes	Pretreated for 24 h before 10 ng/mL IL-1β treatment for 24 h	15, 30, 60 mM	COX-2↓, iNOS↓, PGE2↓, IL-6↓, TNF-α↓	MMP13↓, ADAMTS-5↓	Aggrecan↑, collagen II↑ in a dose-dependent manner	Cytoplasmic IκBa↑, nuclear p65↓ in a dose-dependent manner; nuclear Nrf2↑, cytoplasmic HO-1↑	NF-κB and Nrf2/HO-1	Luo et al. [71]
	Mouse	DMM-induced OA for 8 weeks	50 mg/kg (i.p)	COX-1↑, COX-2↓, mPGES-1↑, mPEFS-2↓	Smoother cartilage surface; loss of proteoglycan↓; OARSI score↓				
Oxymatrine	h-OA chondrocytes	Treated with 1 μg/mL LPS	0.5, 1, 2 mg/mL	IL-6↓, IL-8↓, TNF-α↓	MMP-2↓, MMP-9↓, MMP-13↓		p-p65↓, p-ERK↓, p- JNK↓, p-p38↓	NF-κB and MAPKs	Jiang et al. [72]
	h-OA articular cartilage	Co-treated with 10 μg/mL LPS for 7 or 14 days			GAG release↓; proteoglycan loss↓	Collagen II↑			
	Mouse	ACLT-induced OA for 2 weeks	25, 50 mg/kg (i.p.)		MMP-9 and MMP13-positive chondrocytes↓; destruction of cartilage↓		Cell apoptosis↓, p-65-positive chondrocytes↓		
Hinokitiol	Rat chondrocytes	Pretreated for 2 h before 5 ng/mL IL-1β treatment for 24 h	10, 20, 40, 80 μM		MMP-1↓, MMP-3↓, and MMP-13↓ in a dose-dependent manner	Collagen II↑		Wnt/β-catenin	Li et al. [73]
	Rat	MIA-induced OA for 1 week	20 μL, 80 μM (i.a.)		MMP-1↓, MMP-3↓, and MMP-13↓ in a dose-dependent manner	Mankin score↓			
Tetrandrine	Rabbit chondrocytes	Pretreated for 1 h before 5 ng/mL IL-1β treatment for 24 h	5, 10, 20 μM		MMP-1↓, MMP-3↓, and MMP-13↓	TIMP-1↑	β-Catenin↓	Wnt/β-catenin	Zhou et al. [74]
	Rabbit	ACLT-induced OA for 1 month	6 μg (i.a)		MMP-1↓, MMP-3↓, MMP-13↓; bone wear↓, cartilage degradation↓	TIMP-1↑; Mankin score↓	β-Catenin↓		
Polygalacic acid	Rat chondrocytes	Co-treated with 10 ng/mL IL-1β for 24 h	50, 100 μM	COX-2↓ in a dose-dependent manner	MMP-3↓, MMP-9↓, MMP-13↓ in a dose-dependent manner		β-Catenin↓, p-p38↓, p-ERK↓, p-JNK↓	Wnt/β-catenin and MAPKs	Xu et al. [75]
	Rat	DMM-induced OA for 6 weeks	100 μM (i.a.)	COX-2↓	Mankin score↓				
Icariin	SW1353 cell line	Pretreated for 1 h before 10 ng/mL IL-1β treatment for 24 h	5, 10, 20, 40, 80, 100 μM		MMP-13↓ at 10 𝜇M and 20 μM		p-p38↓, p-p46↓, p-p54↓, β-catenin↓	Wnt/β-catenin and MAPK	Zeng et al. [76]
	Rat	ACLT-induced OA for 6 weeks	20 μM (i.a)		MMP-13↓	Mankin score↓	p-p38↓, P-p46↓, P-p54↓, β-catenin↓		
Zingerone	SW1353 cell line	Treated with 2 ng/mL IL-1β	10, 20, 40 μM	TNF-α↓, IL-6↓, and IL-8↓in a dose-dependent manner	MMP-13↓ in a dose-dependent manner		p-p38↓, p-JNK↓	p38 and JNK/MAPKs	Ruangsuriya et al. [77]
	Porcine cartilage	Treated with 10 ng/mL IL-1β for 5 days or 30 days	10, 20, 40 μM		MMP-13↓, s-GAG release↓, hyaluronic acid release↓	Uronic acid↑, collagen↑			
Geniposide	Rabbit OA chondrocytes	24 h	80 μg/mL	IL-1β↓, TNF-α↓	MMP-13↓	Collagen II↑	p-p38↓	p38/MAPK	Chen et al. [78]
	Rabbit	ACLT-induced OA for 2 weeks	40 mg/kg (i.g.)	IL-1β↓, TNF-α↓, NO↓	MMP-13↓				
Ginsenoside Rb1	SW 1353 cell line	Pretreated for 1 h before 10 ng/mL IL-1β treatment for 24 h	80 μM		MMP-13↓	Collagen II↑	Notch1↓, JAG1↓	Notch	Wang et al. [79]
	Rat	ACLT-induced OA for 6 weeks	0.3 mL, 80 mM (i.a.)		MMP-13↓; cartilage lesions↓	Collagen II↑; Mankin score↓	Notch1↓, JAG1↓		
Halofuginone	Mouse and rat	ACLT-induced OA	1 mg/kg (i.p.)		MMP-13↓, ADAMTS-5↓, proteoglycan loss↓	Collagen II↑, aggrecan↑, lubricin↑; Mankin score↓	Collagen X↓; calcification of articular cartilage↓; aberrant angiogenesis↓ in subchondral bone; subchondral bone remodeling↑; Th17-induced osteoclastic bone resorption↓; p-Smad2↓, pSmad2/3-positive cells↓	TGF-β/Smads	Cui et al. [80]
Celastrol	Rat chondrocytes	Pretreated for 12 h before 10 ng/mL IL-1β treatment for 18 h			MMP-13↓, ADAMTS-5↓			SDF-1/CXCR4	Wang et al. [81]
	Rat	MIA-induced OA for 2 weeks	1, 2 mg/kg (i.a.)		MMP-3↓, MMP-9↓, MMP-13↓, Runx2↓	Collagen II↑, aggrecan↑, proteoglycan↑; OARSI score↓	SDF-1↓ and CXCR4↓ in a dose-dependent manner		
Wogonin	h-OA chondrocytes	Pretreated for 2 h before 1 ng/mL IL-1β treatment for 24 h	10, 25, 50 μM	IL-6↓, COX-2↓, PGE2↓, iNOS↓, NO↓	MMP-13↓, MMP-3↓, MMP-9↓, and ADAMTS-4↓ in a dose-dependent manner	Collagen II↑, aggrecan↑	ROS↓ in a dose-dependent manner; Nrf2↑, HO-1↑, SOD-2↑, NQO-1↑, and GCLC↑; p-ERK1/2↑	ROS/ERK/Nrf2	Khan, et al. [82]
	h-OA cartilage	Pretreated for 2 h before 25 ng/mL IL-1β treatment for 72 h	10, 25, 50 μM		s-GAG release↓; proteoglycan loss↓	Collagen II↑ in a dose-dependent manner			

Anabolic activity is of equal significance since the destruction of the articular cartilage is mainly caused by an imbalance of synthesis and degradation of the extracellular matrix (ECM). Hence, the development of drugs that promote the proliferation of chondrocytes and the formation of ECM, resulting in cartilage regeneration, is another research direction. The small-molecule halofuginone [80] isolated from the plant *Dichroa febrifuga* lowered proteoglycan loss and calcification of articular cartilage in rodents subjected to anterior cruciate ligament transection (ACLT) compared with vehicle-treated ACLT controls. Besides, halofuginone reduced the expression of collagen X, MMP-13, and ADAMTS-5, while increasing lubricin, collagen II, and aggrecan. Wogonin [85] derived from the root extract of *Scutellaria baicalensis* exerted chondroprotective effects through suppression of inflammation/oxidative stress (reducing the expression of IL-6, COX-2, PGE2, iNOS, and NO), matrix degradation (lowered the expression of MMP-13, MMP-3, MMP-9, and ADAMTS-4), and stimulation of collagen II and aggrecan expression in OA chondrocytes and cartilage explants. Ligustrazine [65] protected chondrocytes against IL-1β-induced injury presumably by downregulating the expression of IL-1, IL-6, TNF-α, and MMP-13 and by upregulating the expression of collagen II and aggrecan associated with regulation of SOX9 and inactivation of NF-κB. In human osteoarthritic chondrocytes, epimedin C demonstrated significant anabolic effects by increasing the expression of collagenous and non-collagenous matrix proteins, such as cartilage oligomeric matrix protein, and growth factors, including growth differentiation factor 5 and connective tissue growth factor; both vanillic acid and epimedin C also suppressed the MMP activity in the chondrocyte inflammation model [84].

## Signaling pathways as therapeutic targets

**Fig. 2:**
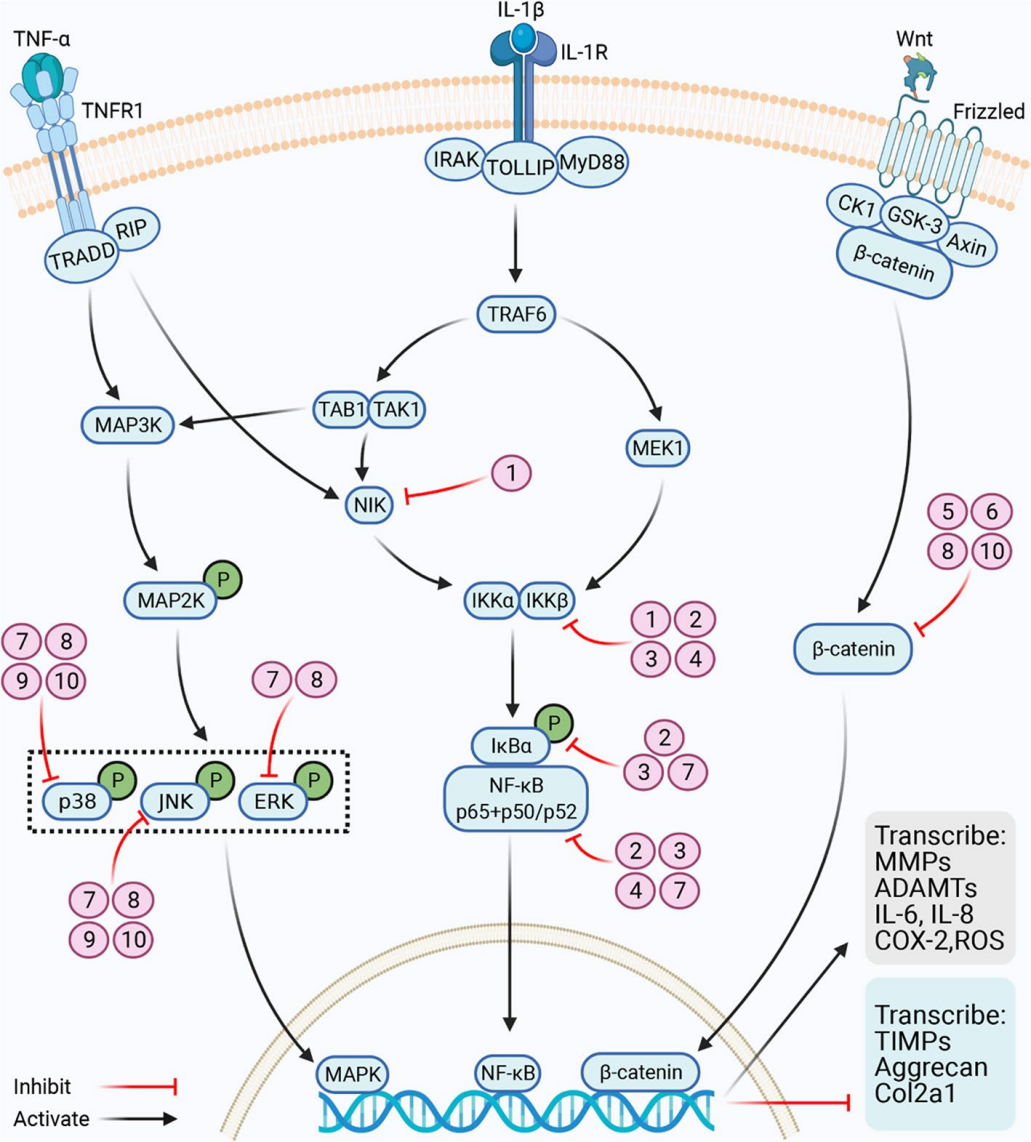
Potential therapeutic targets of small molecules with herbal origin in signaling pathways associated with cartilage degeneration. Name of the compounds: (1) polyphenol-rich pomegranate fruit extract, (2) Egb761, (3) polyoxypregnane glycoside, (4) anemonin, (5) hinokitiol, (6) tetrandrine, (7) oxymatrine, (8) polygalacic acid, (9) zingerone, and (10) icariin. As shown above, some of the compounds have multiple signaling targets. (The figure was prepared with Biorender). TNF-α, tumor necrosis factor-α; TNFR1, tumor necrosis factor receptor 1; TRADD, TNF receptor death domain; MAPK, mitogen-activated protein kinase; IL-1β, interleukin-1β; IL-1R, interleukin-1 receptor; IL-1RAcP, interleukin-1 receptor accessory protein; TOLLIP, Toll-interacting protein; NIK, NF-κB inducible kinase; IKKα, IκB kinase α; IKKβ, IκB kinase β; IκB, NF-κB inhibitor; NF-κB, nuclear factor κB; MMPs, matrix metalloproteinases; ADAMTS, a disintegrin and metalloproteinase with thrombospondin motifs; IL-6, interleukin-6; IL-8, interleukin-8; COX-2, cyclooxygenase-2; ROS, reactive oxygen species; TIMPs, tissue inhibitor of metalloproteinases; Col2a1, collagen 2a1

Generally, with the imbalance between anabolism and catabolism of the articular cartilage, excessive destruction of the ECM results in progressive degeneration. The pathophysiological process of articular cartilage degeneration involves a series of cell signal transduction pathways. Therefore, molecular therapy targeting these signaling pathways is a major research direction (Fig. 2).

### NF-κB signaling pathway

NF-κB is a widely expressed transcription factor family existing ubiquitously in eukaryotic cells, which is associated with the pathogenesis of a number of inflammatory diseases and plays a complex role in different cell types and disease states [86]. The NF-κB family consists of the following five members: RelA (p65), RelB, c-Rel, NF-κB1 (p50 and p105), and NF-κB2 (p52 and p100) [87]. It has been demonstrated that the NF-κB signaling pathway plays a significant role in the course of OA [88]. NF-κB is usually present in the cytoplasm in the form of p56/p50/IκB trimer complexes. Both IκBa and IκBb of the inhibitory IκB protein family are the major regulators of NF-κB activity [89, 90]. Once the cell is stimulated by activating agents, such as cytokines, bacteria/viruses, and stress, IκBα is phosphorylated and degraded rapidly, resulting in the release and nuclear translocation of NF-κB followed by the activation of gene transcription [91].

The NF-κB protein family exists in various cells and is highly expressed in the synovial tissue, cartilage, subchondral bone, joint fluid, and surrounding muscle [92]. NF-κB is involved in almost all pathological processes of OA. Many small molecules of herbal origin can interfere with the development of OA by regulating the NF-κB signaling pathway. Haseeb et al. found that a polyphenol-rich pomegranate fruit extract inhibited the increase in the phosphorylation level of NF-κB p65 in IL-1β induced osteoarthritic chondrocytes [93]. Egb761 extracted from *Ginkgo biloba* reduced p-NF-κB p65 protein level and prevented the activation of the NF-κB pathway in TNF-α induced human chondrocytes [94]. Polyoxypregnane glycoside from *Dregea volubilis* extract inhibited the IL-1β-induced expression of MMPs via the suppression of NF-κB in human chondrocytes [95]. Anemonin showed anti-inflammatory, anti-catabolic, and anabolic effects on IL-1β-induced human chondrocytes in vitro, human cartilage explants ex vivo, and in a destabilization of the medial meniscus (DMM)-induced mouse OA model through the suppression of the IL-1β/NF-κB pathway activation [58]. Similarly, RNA sequencing analysis revealed the interference of vanillic acid with NF-κB signaling in human chondrocytes under inflammatory conditions [84].

### Wnt/β-catenin signaling pathway

Elevated expression of Wnt/β-catenin has also been observed in OA [96, 97]. Activation of the Wnt/β-catenin signaling pathway can increase the expression of MMPs and aggrecanases (ADAMTS-4 and ADAMTS-5) [98, 99], leading to the degradation of the ECM, which in turn results in the development of OA [100]. However, inhibition of the β-catenin signaling may also cause defects in postnatal cartilage development or even cartilage destruction [101, 102], suggesting that the Wnt/β-catenin signaling pathway may play a dual role in the pathogenesis of OA. To develop effective small molecules for the treatment of OA, the precise regulation of the Wnt/β-catenin signaling pathway should be considered critically.

Hinokitiol is a natural tropolone-related compound found in the heartwood of *Cupressaceae* plants that has a wide range of biochemical and pharmacological activities, including anti-bacterial [103], anti-tumor [103], and antioxidant capacities [104]. In addition, Li et al. found that hinokitiol also reduced MMP expression by inhibiting Wnt/β-catenin signaling in vitro and in vivo [73]. Tetrandrine purified from the root of *Stephania tetrandrine* of the *Menispermaceae* family was also found to alleviate OA pathways in vitro and in vivo via inhibiting Wnt/β-catenin signaling [74].

### MAPK signaling pathways

Three MAPK families have been comprehensively characterized, namely extracellular signal-regulated kinase (ERK), C-Jun N-terminal kinase (JNK/SAPK), and p38 Kinase, which is closely related to the pathogenesis of OA [105]. The anti-inflammatory and anti-catabolic effects of oxymatrine and polygalacic acid were mediated via the inhibition of the phosphorylation of ERK, JNK, and p38 related to the MAPK pathways [72, 75]. Zingerone suppressed cartilage degradation by involving the p38 and JNK MAPK signaling pathways [77]. Icariin showed an anti-catabolic effect via inhibiting the phosphorylation of p38 in IL-1β-induced human SW 1353 chondrosarcoma cells and a rat OA model [76].

### Other signaling pathways

AMP-activated protein kinase (AMPK), a crucial regulator of energy metabolism, is a heterotrimeric serine/threonine protein kinase comprising a catalytic α-subunit and two regulatory β- and γ-subunits [106, 107]. AMPK is activated by a conserved threonine (THr172) phosphorylation in the α-subunit as a response to decreased cellular AMP/ATP ratio [106, 108]. Activated AMPK is constitutively expressed in normal articular cartilage, while its level is decreased in OA cartilage due to de-phosphorylation caused by either biomechanical injury or inflammatory cytokines like IL-1β and TNF-α [107, 109]. Natural small molecules like butein and quercetin have shown chondroprotective effects via activation of AMPK [110, 111].

Once activated by extracellular molecules, phosphatidylinositol 3-kinase (PI3K) generates phospholipids, activates downstream protein kinase B (Akt), and further phosphorylates mammalian target of rapamycin (mTOR). It has been proved that the PI3K/AKT/mTOR signaling pathway is involved in cartilage degeneration by affecting its extracellular matrix homeostasis, promoting chondrocytes’ inflammatory response, and especially by inhibiting chondrocyte autophagy [112]. Active compounds, extracted from plants such as icariin and β-ecdysterone, have been demonstrated to alleviate OA by activating autophagy through the regulating PI3K/AKT/mTOR signaling pathway [113, 114]. The PI3K/AKT/mTOR and AMPK signaling pathways have interactions via the common downstream target mTOR; however, they own opposed effects on nutrient and energy homeostasis and cell growth [115, 116].

## Clinical translation and its challenges

Based on recent search results on studies registered with www.clinicaltrials.gov (02.2021, filter keywords: osteoarthritis, drug), only 3 trials (NCT03375814, NCT03715140, NCT02905799) on small molecules from herbal origin entered clinical research, although there have been many TCM formulas or decoction products tested in clinical trials. Resveratrol decreased advanced glycation end product (AGE)-stimulated expression and activity of MMP-13 and prevented AGE-mediated destruction of collagen II through inhibiting IKK-IκBα-NF-κB and JNK/ERK-AP-1 signaling pathways [59]. It is currently in a phase III clinical trial (NCT02905799) for patients with knee OA.

There are several hurdles to overcome in the translation of herbal compounds into clinics. Firstly, the traditional treatment for OA including regular oral, topic, and intra-articular administration confronts the shortfalls of low bioavailability and severe side effects due to the low stability or solubility of small molecules in serum or synovial fluid. Novel delivery systems need to be introduced to deliver the small molecules to the target site and improve their stability under storage conditions and their bioavailability in vivo. Using exosomes with the target specificity as a delivery vehicle, the anti-inflammatory activity of curcumin on inflammatory cells was enhanced with therapeutic, but not toxic, effects [117]. The synergistic effect of promotion of chondrocyte autophagy via exposure to sinomenium encapsulated by chitosan microspheres and photo-crosslinked gelatin methacrylate hydrogel retarded the progression of surgically induced OA [118]. A combination of celecoxib-loaded liposomes and hyaluronate gel via intra-articular injection was more effective than a single drug in pain control and cartilage protection [119].

Secondly, the quality of raw herbs varies and is difficult to standardize, resulting in a great variation in the content of the main ingredients even with regulated extraction procedures. Thirdly, insufficient representative animal models and preclinical experiments make the in vivo evidence deficient. Fourthly, issues related to pesticide and heavy metal residues have been reported [120]. Fifthly, although there are thousands of compounds identified in medicinal herbs, most of them will be disregarded because of unidentified beneficial effects or amount, while few are determined to have definite pharmaceutical effects. Lastly, certain herbs have potentially toxic effects on the liver and kidneys. For instance, berberine and coumarin have potential hepatotoxicity, and β-escin and aristolochic acid may cause nephrotoxicity [121]. Standardized and comprehensive toxicity studies will be necessary to verify the safety of the compounds for clinical application.

## Discussion

The management of OA is unsatisfactory for a significant number of patients who neither respond to the existing conservative treatment nor achieve indications of joint replacement surgery. These include older patients (> 55 years of age) with moderate or younger patients with moderate to severe signs and symptoms of joint degeneration [122]. For these cases, small molecules of herbal origin could represent a promising approach to halt, delay, or even reverse the degenerative process. Indeed, there are several advantages of herbal small-molecule compounds [123, 124]. Firstly, many herbal compounds have the advantages of little side effects [124] making them suitable for long-term treatment [125]; nevertheless, the safety profile of each individual drug needs to be carefully evaluated. Secondly, there is evidence that several compounds have multi-target effects [123], including anti-inflammatory, anti-apoptotic, anti-catabolic, antioxidant, anabolic, and proliferative effects (Table 1). It is conceivable that a combination of potent compounds will have the broadest effect, which may be attractive as an either alternative or complementary treatment to conventional measures [126]. In addition, small molecular compounds of herbal origin have plenty of resources and are generally cost-effective to produce [127].

Nevertheless, despite the positive outcome of preclinical in vitro and in vivo studies, translation towards clinical application is still in its early stage. There are several reasons for this slow-going progress in drug development. In preclinical models, the pathogenesis and progression are standardized and reproducible, while the heterogeneity among patients makes their responses greatly variable. Therefore, it will be important for future clinical studies to classify patients according to their OA stages and phenotypes, which is an emerging field in clinical research [128]. Factors to consider include previous joint trauma, comorbidities like metabolic disorders, clinical manifestations, and eventually the genetic and epigenetic background. Both the limitation of preclinical models and the failure to clinically diagnose OA at the early stage hamper the development of effective therapies.

The delivery of the compounds also needs to be carefully evaluated. Unlike typical biologics, small molecular drugs are in principle suitable for oral application; however, it needs to be evaluated whether the required activity at the joint site can be achieved. On the other hand, the joint space appears to be suitable for intra-articular application [129]. Novel drug delivery systems have been developed to facilitate controlled drug release in intra-articular applications [130]. In the described animal studies, the compounds have primarily been applied intra-articularly, while intraperitoneal and intragastric delivery was also reported (Table 1). This suggests that systemic application may be sufficiently effective for the respective compounds. Topical application in the form of penetrating formulations can also be considered, although it is more challenging to standardize and improve the dosing into the joint. Interestingly, there is evidence from clinical trials for topical comfrey extract [131] as an analgetic treatment in musculoskeletal pain including OA. However, more rigorous, high-quality controlled studies need to be performed to confirm the therapeutic benefit.

## Conclusion

In conclusion, small-molecule compounds of herbal origin may have significant potential for the treatment of OA. Advanced techniques are available for efficient isolation and purification of small molecular compounds from herbs. Numerous preclinical studies have elucidated the mechanisms of action and identified the signaling pathways modulated by the individual compounds. For future studies, patient stratification will be essential because of the heterogeneity among people with OA. The therapeutic benefit may be most pronounced if the compounds can be applied at the early stage of OA, when tissue structures are still intact and functional responsive cells are still present in the joint. Controlled clinical trials of high quality will be needed to confirm the beneficial effects demonstrated in the preclinical studies.

## Data Availability

All data are included in this article.

## Abbreviations

SOD: Superoxide dismutase
MDA: Malondialdehyde
DMM: Destabilization of the medial meniscus
MIA: Monoiodoacetate
ACLT: Anterior cruciate ligament transection
SW 1353 cell line: Human SW 1353 chondrosarcoma cells
LPS: Lipopolysaccharides
i.a.: Intra-articular
i.p.: Intraperitoneal
i.g.: Intragastric
AGEs: Advanced glycation end products
AP-1: Activator protein-1
mPGES: Microsomal PGE synthase
h-: Human
OA: Osteoarthritis
MMP: Matrix metalloproteinase
ADAMTS: A disintegrin-like and metalloproteinase with thrombospondin motifs
GAG: Glycosaminoglycan
